# Appendiceal Goblet Cell Adenocarcinoma Case Report and Review of the Literature

**DOI:** 10.7759/cureus.13511

**Published:** 2021-02-23

**Authors:** Kevin Sigley, Michael Franklin, Stephen Welch

**Affiliations:** 1 General Surgery, Beaumont Health, Dearborn, USA; 2 Pathology, University of New Mexico, Albuquerque, USA; 3 Surgery, Beaumont Health, Dearborn, USA

**Keywords:** carcinoma of the appendix, appendix, goblet cell adenocarcinoma, appendectomy, right colectomy, hemicolectomy, oncology, surgery, goblet cell carcinoid, cdx2

## Abstract

Acute appendicitis is a common presentation to the emergency department. The common pathogenesis thereof relates to obstruction of the appendiceal lumen by an appendicolith, which leads to an increase in intraluminal and intramural pressure. This is followed by distension of the appendix, subsequent small vessel occlusion and lymphatic stasis, and appendiceal wall ischemia and necrosis, eventually leading to rupture if not treated. Occasionally tumors at the base of the appendix can lead to appendicitis via the same process as an appendicolith. Goblet cell adenocarcinoma (alternatively named goblet cell carcinoid) is amongst the most rare appendiceal tumors, with a reported incidence rate of 0.05 cases per 100,000 population per year in the United States. These tumors contain features of both neuroendocrine tumors as well as adenocarcinomas, but behave more similarly to adenocarcinomas. Consensus regarding management of these tumors is lacking, likely due to the rarity of the disease. In this paper, we present a case of appendiceal goblet cell adenocarcinoma causing appendicitis and review the literature regarding these rare epithelial tumors.

## Introduction

Appendiceal goblet cell adenocarcinoma (alternatively called goblet cell carcinoid [GCC]) is a rare diagnosis, with 369 cases reported from the SEER database from 1973 to 2001, corresponding to an age-adjusted 0.05 cases/100,000 population per year [1]. The most common presentations are chronic abdominal pain, appendicitis, and abdominal distention, but these are also frequently found incidentally at appendectomy. Goblet cell adenocarcinomas exhibit positive staining for neuroendocrine markers like a traditional well-differentiated neuroendocrine tumor (NET) (carcinoid tumor), but are recognized to behave more aggressively like conventional adenocarcinomas. The tumor, node, metastasis (TNM) system is used for staging, and prognosis depends on grade of tumor and stage at diagnosis. Because these tumors are commonly discovered and then staged on pathology analysis, the main surgical question is whether additional surgery (i.e. right hemicolectomy for lymph node evaluation) is required for staging and/or treatment. There is not a consensus on optimal management strategy due to the rarity of the disease. In this report, we present a case of appendiceal goblet cell adenocarcinoma causing appendicitis and review the current management recommendations.

## Case presentation

A 57-year-old male presented to the emergency department complaining of right lower quadrant abdominal pain that had migrated from the periumbilical region. He did complain of nausea but denied any other symptoms including fever, vomiting, diarrhea, constipation, and urinary changes. The patient had no prior surgical history. He took lisinopril for hypertension but no other medications. There was no pertinent family history. The patient had undergone a screening colonoscopy at age 50, which was without abnormality.

Physical examination revealed an obese middle-aged male in mild pain distress, with blood pressure of 134/87 mm Hg, pulse of 89 beats/minute, oral temperature of 98.4°F (36.9°C), respiratory rate of 18 breaths/minute, SpO_2_ of 96%, and a BMI of 37.11 kg/m². The abdomen was obese and soft, with focal peritonitis in the right lower quadrant, positive rebound tenderness and guarding to palpation. Auscultation of the heart and lungs revealed a regular rate and rhythm without murmur and clear breath sounds.

**Figure 1:**
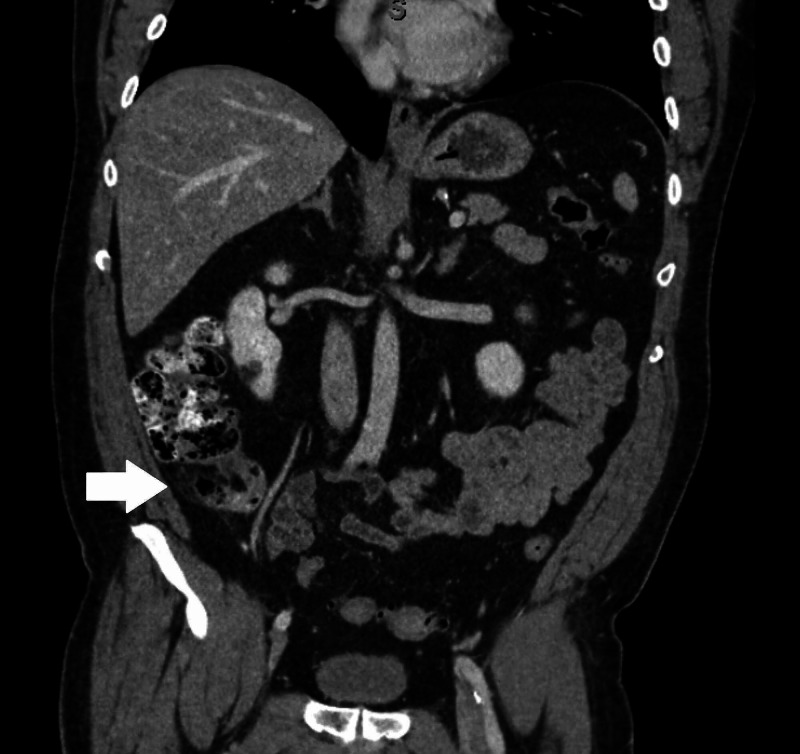
Coronal view of CT showing dilated and inflamed appendix.

He underwent CT scanning of the abdomen, which revealed evidence of acute appendicitis, with a distended 1.6-cm-diameter appendix, surrounding edema, and partial obstruction with fecalization of the contents of the appendix (Figure 1). Laboratory evaluation was also undertaken, which revealed a mild leukocytosis to 14.7 thousand/µL with left shift (neutrophils 84%).

**Figure 2:**
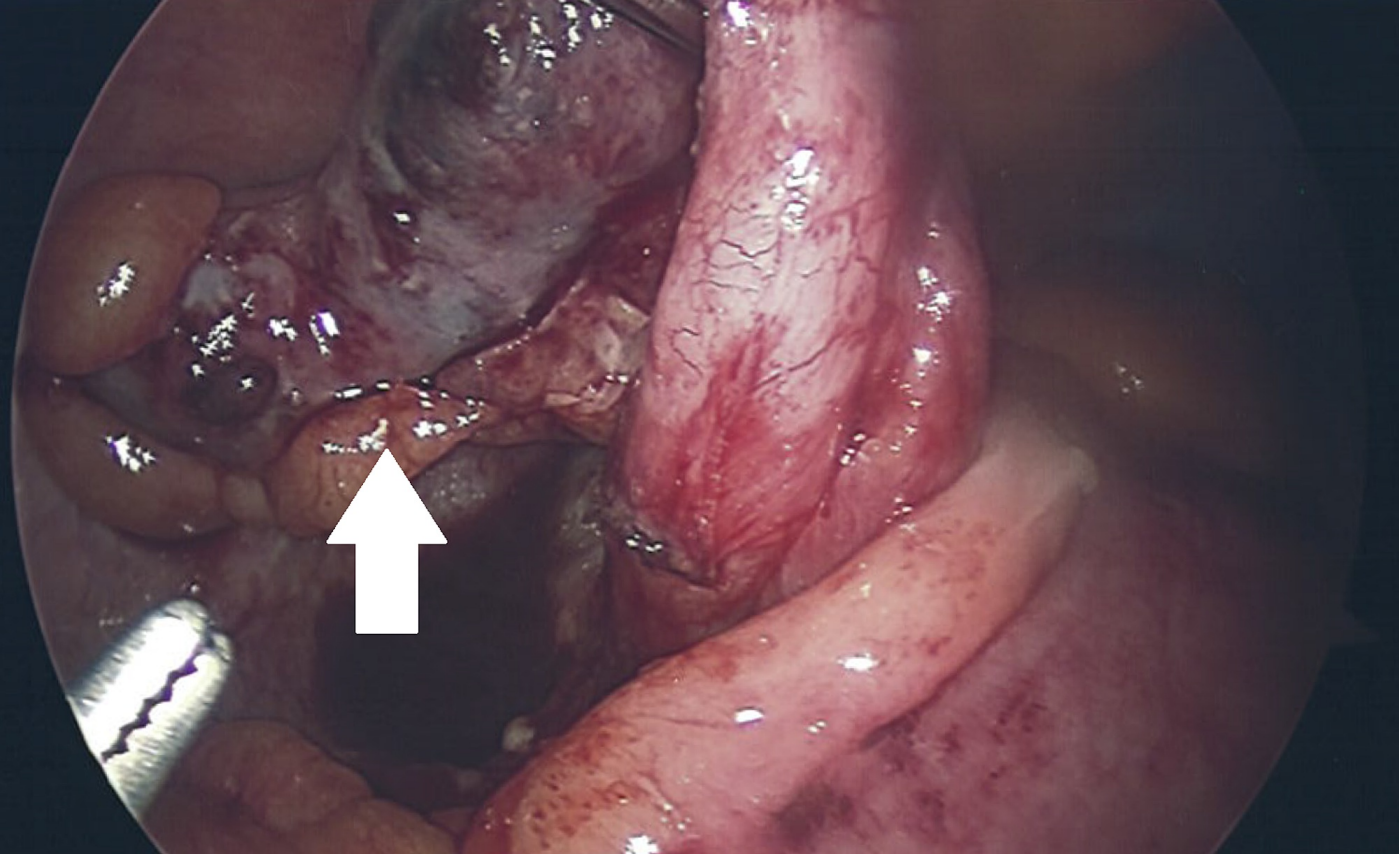
Intraoperative laparoscopic photo showing inflamed appendix after dissection from abdominal sidewall and cecum.

Given the CT findings in conjunction with the patient’s symptoms and laboratory findings, the patient was taken to the operating room for laparoscopic appendectomy. Intraoperatively, inflammation and adhesions were apparent in the right lower quadrant. The appendix was retrocecal, adherent to itself and to the lateral abdominal wall. Careful blunt dissection was undertaken, freeing the appendix from its adhesions. There was an area of gangrene without perforation in the middle of the appendix. There was no perforation or abscess (Figure 2). There was a small amount of purulence surrounding the appendix. Using the Harmonic® endoshears (Ethicon, Somerville, NJ, USA), the mesoappendix was divided and hemostasis was obtained. Next, using a 45 mm GIA™ (Medtronic, Minneapolis, MN, USA) stapler with blue load, the appendix was clamped, stapled, and divided at the base of the appendix, including a small portion of the cecum. The appendix was then removed using an Endo Catch™ (Medtronic, Dublin, Ireland) bag.

**Figure 3:**
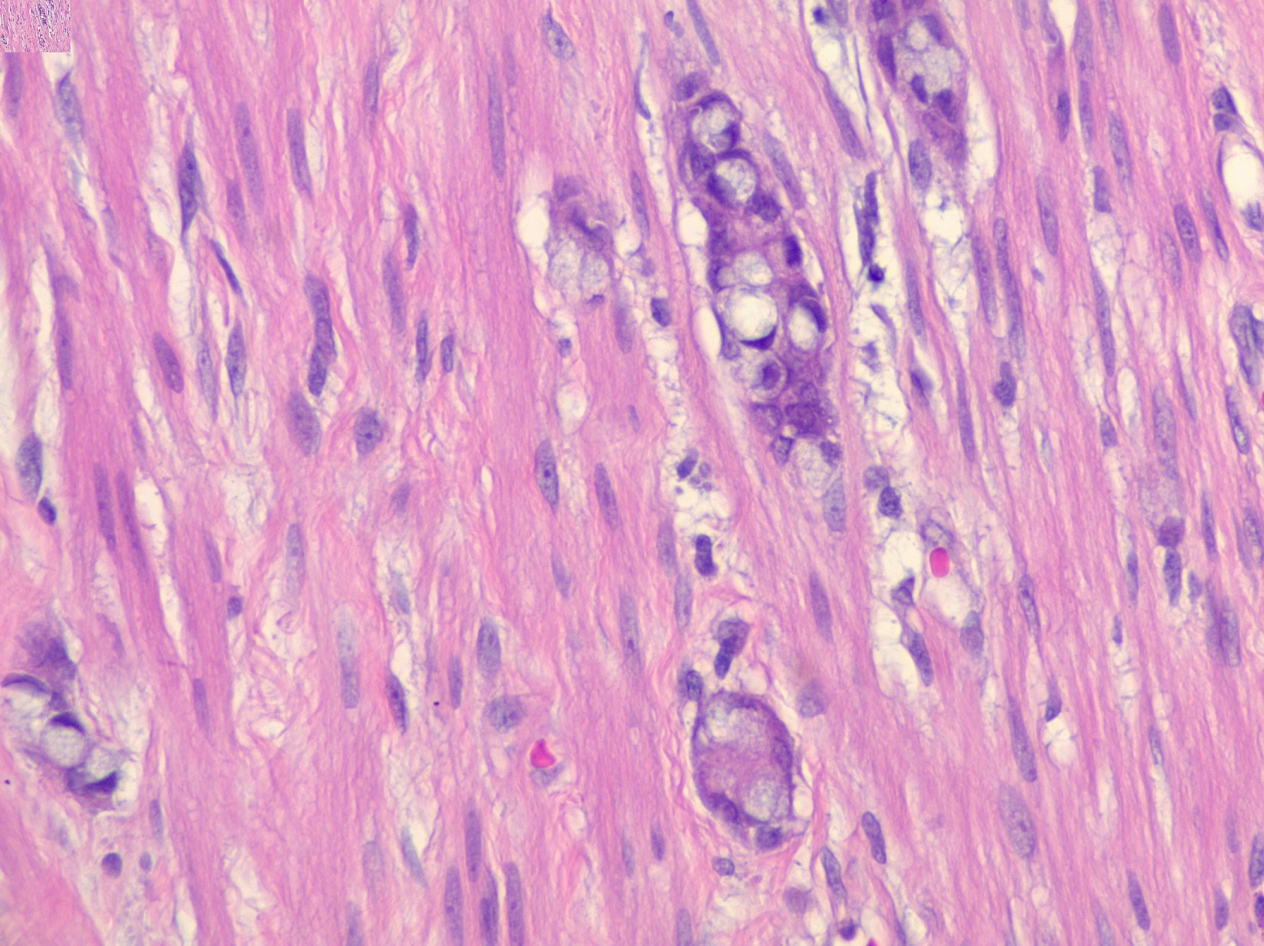
High power view of well-to-moderately differentiated goblet cell adenocarcinoma.

**Figure 4:**
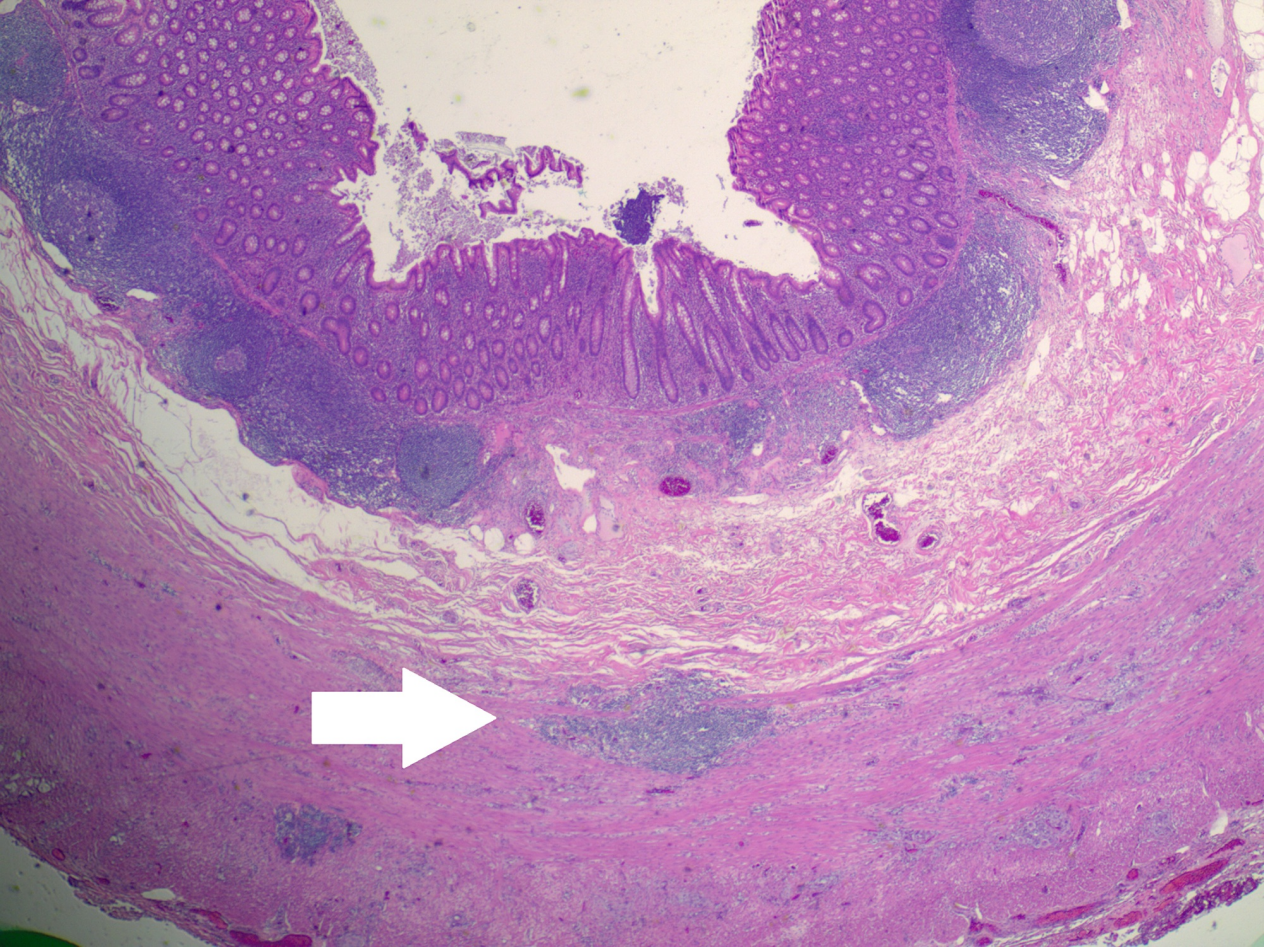
Low power view of goblet cell adenocarcinoma invading muscularis propria of the appendix.

**Figure 5:**
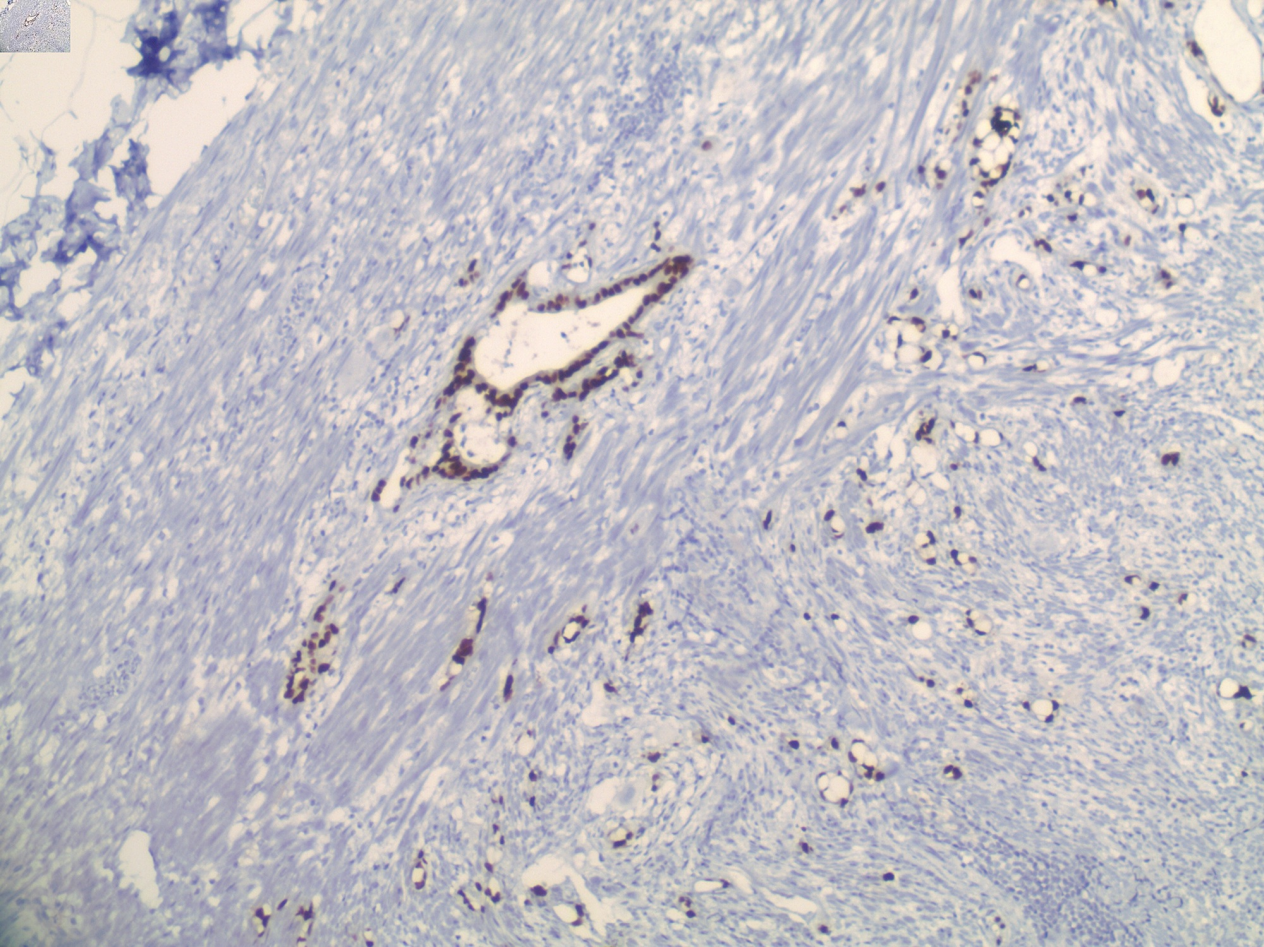
Goblet cell adenocarcinoma with positive CDX2 immunostain.

Pathologic analysis of the specimen revealed a well-to-moderately differentiated 1.5 cm goblet cell adenocarcinoma (Figure 3), with associated acute appendicitis, with the tumor extending to the proximal margin. The tumor extended through the muscularis propria into periappendiceal adipose tissue (Figure 4), but did not appear to involve the serosal surfaces. There was no lymphovascular invasion. The neoplastic cells stained positive for CDX2, indicating adenocarcinoma cells of intestinal origin (Figure 5). Synaptophysin was focally positive. The specimen was signed out as goblet cell adenocarcinoma, pathologic stage (AJCC 8th Edition): pT3 Nx Mx.

Postoperatively, the patient was evaluated by oncology with a plan for further staging. However, the patient experienced prolonged ileus, and then on postoperative day #9, he developed a lower GI bleed, suspected to be a delayed bleed from the tumor in the staple line, requiring a total transfusion of five units packed red blood cells. He underwent bowel preparation for a colonoscopy to investigate his GI bleed but developed large volume hematochezia and hemodynamic instability, so was taken to the OR emergently for exploratory laparotomy. He underwent right hemicolectomy with high ligation of the right colic and ileocolic pedicles for lymph node evaluation, and end ileostomy creation due to hemodynamic instability. Pathology analysis of this specimen revealed periappendiceal abscess, but no evidence of goblet cell adenocarcinoma, with seven lymph nodes negative for carcinoma.

## Discussion

The usual pathogenesis of appendicitis is obstruction of the appendiceal lumen, which leads to increase in intraluminal and intramural pressure, which leads to small vessel occlusion and lymphatic stasis, distension of the appendix, appendiceal wall ischemia and necrosis, and eventually perforation if not treated [2]. This report describes a case of a goblet cell adenocarcinoma causing acute appendicitis found on pathology examination of the specimen. Although the proximal margin was positive, there was no evidence of adenocarcinoma on right hemicolectomy, and no positive lymph nodes from the seven in the specimen. The decision to proceed with right hemicolectomy in this case was made due to a positive proximal margin, as well as a GI bleed likely due to the tumor in the staple line, but the consensus regarding criteria for completion hemicolectomy after diagnosis from appendectomy is lacking.

Goblet cell adenocarcinoma was previously known as GCC or adenocarcinoid, as it has features of both adenocarcinoma and NET. However, they are more aggressive than typical well-differentiated NETs of the appendix and are classified and staged as appendiceal adenocarcinomas. The mean patient age of goblet cell adenocarcinoma is 52 years. Men and women are affected equally. There are no known risk factors, although some cases of goblet cell adenocarcinoma in China have been associated with schistosomiasis [3].

Staging of goblet cell adenocarcinoma is similar to that of other colorectal adenocarcinomas due to their more aggressive nature than NETs. The TNM nomenclature is used: T1 tumors extend into the submucosa, T2 tumors invade into the muscularis propria, T3 tumors invade the subserosa/mesoappendix, and T4 tumors extend into the peritoneum/other organs. In contrast, NETs of the appendix are staged primarily by size. N0 indicates no nodal involvement, N1 indicates one to three positive regional lymph nodes, and N2 indicates four or more positive regional lymph nodes. M0 indicates no distant metastases, and M1 indicates distant metastases [4].

Prognosis is based on stage at diagnosis and grade of tumor. In a retrospective analysis, the five-year overall survival rate for stages I, II, III, and IV were 100%, 76%, 22%, and 14%, respectively [5]. Stage of tumor also seems to relate to the likelihood of positive lymph nodes: 28% of patients with T4 tumors had positive lymph nodes, while none of the patients with T1-3 tumors did. In another study, the performance of hemicolectomy did not impact relapse rate or disease-free survival [6]. Histologic grade also correlated with overall survival, independent of tumor stage, with median overall survival of 204, 86, and 29 months for low-grade, intermediate-grade, and high-grade goblet cell adenocarcinomas, respectively [7]. A commonly used pathologic grading system was proposed by Tang in 2008, at which time these tumors were called GCC, and were divided into two groups: typical GCCs are deemed as Tang "class A" tumors and exhibit well-defined goblet cell morphology without significant atypia. The second group was called adenocarcinoma ex-GCC on the basis of the histologic features of the tumor at the primary site. The second group is further divided into signet ring cell type (group B) and poorly differentiated adenocarcinoma type (group C). The stage IV-matched five-year survival was 100%, 38%, and 0% for groups A, B, and C, respectively [8].

Some authors advocate for right hemicolectomy for all appendiceal goblet cell adenocarcinomas, some believe this is only necessary for T3 or T4 tumors, and others advocate for right colectomy only if the tumor is >2 cm in size, is poorly differentiated, involves the base of the appendix, is associated with nodal metastases on imaging, or has high-grade histologic features. The role of adjuvant therapy for patients who have early-stage goblet cell adenocarcinoma is unknown, but in the setting of node-positive (stage III) disease or higher, adjuvant chemotherapy is recommended. In a study of 57 patients, Pham found no significant difference in five-year survival in patients with stages II, III, and IV disease who underwent simple appendectomy versus right hemicolectomy (43% and 34%, p=0.604), as well as no significant difference in those who received adjuvant chemotherapy and those who did not (32% and 27%, p=0.151) [5]. However, the scarcity of the disease may limit generalizability.

The most common site of metastasis is the peritoneum, which can be managed with cytoreductive surgery and heated intraperitoneal chemotherapy (HIPEC). In a study from 2004, the overall median survival in 22 patients with peritoneal carcinomatosis due to goblet cell adenocarcinoma treated with cytoreductive surgery and HIPEC was 18.5 months (range 3-95) [9]. A recent Swedish study showed even better long-term survival with a median survival of 30 months (range 9-38 months), a one-year survival rate of 80% and three-year survival rate of 20% [10].

## Conclusions

Goblet cell adenocarcinoma of the appendix is a rare disease process, which can present with chronic abdominal pain, acute appendicitis, or no symptoms at all. Previously classified under the carcinoid umbrella, these tumors display adenomatous as well as neuroendocrine features. The diagnosis is usually made after appendectomy, and the main surgical question is whether the patient requires the right hemicolectomy for further staging and/or treatment. Some authors advocate for right hemicolectomy for all appendiceal goblet cell adenocarcinomas, some believe this is only necessary for T3 or T4 tumors, and others advocate for right colectomy only if the tumor is >2 cm in size, is poorly differentiated, involves the base of the appendix, is associated with nodal metastases, or has atypical histologic features. The prognosis for the tumors directly relates to the stage and grade of the tumor, so accurate staging and pathological examination is critical in the treatment of these rare tumors. As more data regarding the relationship between grade, stage and treatment strategies becomes available and analyzed, more standardized treatment strategies may be developed.
